# Personal Experiences on the Macedonian Front

**Published:** 1919-03-29

**Authors:** 


### MALARIA AND THE ARMY.

### Personal Experiences on the Macedonian Front.

During the last few years few diseases have given rise to more controversy than malaria, but at the moment insufficient time has elapsed for full reports from the various theatres of war to fully co-ordinate opinion and produce a complete survey of the infection in all its manifestations. A most interesting volume* comes from the pen of Captain A. Cecil Alport, R.A.M.C. (T), describing many months' work and experience in hospitals at Salonica, and although, as the author states, the expressed opinions may not find general acceptance, he writes of things exactly as he found them. The book is, therefore, of an essentially practical type, and one can realise the interest it must hold for those who have come into contact with the disease in the same or other of the war areas. Many of the points made are well worthy of brief description. In Macedonia the disease appears in its severest form, probably due to the fact that *P. falciparum*, the most virulent of all malarial types, is particularly common in these lands, a maximum incidence occurring in September, October, and November. Swamps are a common geographical feature, and the natives act as chronic carriers of the gametocytes. Here, as elsewhere, the anopheline mosquitoes are given the entire credit for carrying the disease, and the types culex and stegomyia, though common, are limited to dengue fever. It is well known that the first of these has always been present in certain swampy districts of Great Britain, and the return of very large numbers of soldiers with the parasites in their blood has already led to more than one serious outbreak in England.

### Prophylaxis.

The use of prophylactic quinine appears to have been an absolute failure with the Salonica Force. In most cases of infection treated in 1917 it was found that they had received the 15 grains bi-weekly, and the opinion is given that even much greater doses would have been equally ineffective, doses which would, in fact, have made the prevention worse than the disease. Mosquito-proof houses and huts were valuable, but the extreme complications necessary to produce absolute safety only tended to increase the possibility of wasted expense and effort. Doors ajar, the wear and tear in a war area, and numberless constantly occurring breaks in continuity produced many possible entries for the mosquito. Personal protection was much better; sleeping under efficient nets, and great care to avoid being bitten ranked second only to the soundest way of all, namely, direct attack of the mosquito by drainage systems. The technique of these measures is so widely understood that in this particular country their accomplishment and success calls for no special comment, but the details of "oiling" the surface of pools is interesting, if only for its simplicity. Removal of vegetation, paraffin spraying down wind of all open water, even tanks, buckets, etc., or the simple drip from a can permanently supported over a slowly running stream were all employed. In the hot months all possible troops were withdrawn to camps in the hills, where there were comparatively few mosquitoes, many nets rendered non-inflammable by ammonium chloride provided, and all brushwood within a quarter mile of the camp cut down and burned. The following paragraph describes the usual situation which has to be met and surmounted.

> Mosquitos only bite when it is hot. In the winter they do not suck blood at all, but on the Struma they commence as early as February. On a warm sunny day at this time of year they will bite at noon. As the summer advances their period of activity becomes later and later. By May they are found to be biting in the early morning and evening only while at the height of the hot season, July and August, they are busy all night. This activity gradually decreases through the autumn, until the winter arrives and they become innocuous and so the cycle goes on.

The author makes one criticism. Much of the work is left until late spring, when it could be well advanced during the winter months. Also many of the men in the country had been already infected before prophylaxis developed, and the treatment is unfortunately not conducted on the same natural and thorough lines as the work of disease prevention.

### Points in Treatment.

A strong protest is to be made against the use of quinine in tablets. They are frequently recovered apparently unchanged from the stools of the patients. Solutions are better and readily absorbed. Quinine bihydrochloride, although expensive, is very soluble in water, and has been found the best drug to use either by mouth, rectum, intramuscularly or intravenously. It is absolutely essential to bring quinine into the system at the first possible moment; hence the value of the latter methods, and parallel to this fact comes the necessity of using much larger doses of quinine than in past years. The amount of drug injected must be controlled by the reaction to it of the disease, and short of actual quinine poisoning the administration should be, if necessary, pushed to its utmost limits. The author is strong in producing evidence in support of the view that had greater doses been employed, the mortality would have been considerably reduced.

Malarial toxins can produce almost any disturbance of the system, and it must be realised that, though the combat within the patient may be coincident with great nearness to his death, quinine, if in sufficient concentration, is more than a match for the parasite.

### Chronic Malaria.

Experience gained in this area with treatment after the acute stage has passed proves the much greater resistance of the malignant variety. Crescents of this parasite are particularly hard to kill,

* *Malaria and its Treatment.* By Captain A. Cecil Alport, R.A.M.C. (T). (Bale Sons & Danielsson, 21s. net.)

**Malaria and the War**—(*continued*).

and quinine in almost any doses has no effect upon them. Therefore it becomes necessary to saturate the system with the drug to forestall and destroy the young trophozoites, etc., whenever sporulation takes place. The amount of quinine is controlled by the frequency or tendency to relapse and, generally speaking, any backward tendency is strong indication that administration must be pushed further up to about 60 grains a day for a week, after which, if improvement occurs—as in the author's experience it usually does—a gradual reduction to 30 grains a day is made, and this strength maintained until the spleen is normal and no relapse has occurred for at least three months. Only severely malignant types resist this treatment, and even then an additional course of arsenic, with the further quinine administration, is usually sufficient.

Arguments against large doses of quinine are most emphatically refuted. Giddiness and deafness pass off, or may be treated with bromides. The general condition rapidly improves with decline of the malarial toxæmia, and much of the blame for low vitality should be attached to the disease rather than the drug. That anæmia is directly caused is stated to be an absolute fallacy, and many blood counts are given in evidence, and considering the known destruction of corpuscles by the parasites the figures read very convincingly.

BLACKWATER FEVER.

A careful distinction must be drawn between the common malarial complication in which a certain amount of hæmoglobin is passed in the urine, and which rapidly disappears with the exhibition of quinine and the above-named condition. True blackwater fever is accompanied by jaundice, green vomit, a very dirty tongue, and is a far more serious complication. Both spleen and liver are enlarged, and the urine, at the worst stages, an almost black porter colour. An acute toxæmia leads to widespread degeneration of the epithelium in the convoluted tubules of the kidney, often of sufficient extent to produce actual suppression of urine. The earlier and more vigorously a case is treated the better. Quinine and injection of saline or Bayliss' alkaline-gum solution to raise pressure in the systematic circulation are suggested, and although a variety of theories are advanced to explain the condition, the author expresses the opinion that it is entirely due to inefficiently counteracted malignant malarial toxins, and is strong in urging the use of quinine in its treatment.

RECORDS IN OTHER WAR AREAS.

From other theatres come very similar accounts of the disease, but, as might be expected, there is considerable divergence of opinion as to the most efficient methods of treatment. On the whole there is a tendency to approve of the increased quinine dosage, and the introduction of galyl and similar injections has led to considerable success in the anæmic and debilitated conditions arising late in the infection. Most of the difficulties lie in the direct attack upon the mosquito haunts themselves, and the ingenuity displayed has been wonderful. Such work is a curious combination of the duties of the sanitary engineer and the specialised scientist, and the greatest credit is due to the many members of the R.A.M.C. who have risen to the tackling of this problem. Natives appear, in a great number of instances, to act as carriers of infection, and although they are themselves severely attacked in epidemic years, yet the members of the Expeditionary Forces have always been the more ready victims. The total number of army infections is tremendous. Already one has referred to the presense of anopheles in England, and it seems we are likely to be faced with a problem for serious consideration if these men and the mosquitoes come into frequent and seasonally correct contact. Captain Alport in no way underestimates the urgency of the situation when he speaks strongly for the more efficient development of general measures aiming at absolute cure of overseas cases, and the greatest care that men, with parasites still possibly alive in their systems, shall not be returned haphazard to this country.

